# Regulatory Roles of Quercetin in Alleviating Fructose‐Induced Hepatic Steatosis: Targeting Gut Microbiota and Inflammatory Metabolites

**DOI:** 10.1002/fsn3.4612

**Published:** 2024-12-20

**Authors:** Jinjun Li, Zhiqi Zhao, Yixuan Deng, Xinxin Li, Liying Zhu, Xin Wang, Li Li, Xiaoqiong Li

**Affiliations:** ^1^ State Key Laboratory for Managing Biotic and Chemical Threats to the Quality and Safety of Agro‐Products and Institute of Food Sciences Zhejiang Academy of Agricultural Sciences Hangzhou China; ^2^ Institute of Food Science, Zhejiang Academy of Agricultural Sciences Hangzhou China; ^3^ Key Laboratory of Postharvest Preservation and Processing of Vegetables (Co‐Construction by Ministry and Province) Ministry of Agriculture and Rural Affairs Hangzhou China; ^4^ School of Medicine Wenzhou Medical University, Chashan University Town Wenzhou Zhejiang China; ^5^ Clinical Medical College, Hangzhou Normal University Hangzhou China

**Keywords:** fructose, gut microbiota, hepatic steatosis, quercetin

## Abstract

While fructose is a key dietary component, concerns have been raised about its potential risks to the liver. This study aimed to assess quercetin's protective effects against fructose‐induced mouse hepatic steatosis. Thirty‐two male C57BL/6J mice were randomly allocated into four groups: control, high fructose diet (HFrD), HFrD supplemented with low‐dose quercetin (HFrD+LQ), and HFrD supplemented with high‐dose quercetin (HFrD+HQ). Biochemical, pathological, immune, and metabolic parameters were assessed. Quercetin treatment significantly reduced liver fat percentages in mice on a high fructose diet, with the most notable reduction observed in the HFrD+HQ group. Histological examination confirmed this reduction, revealing diminished lipid droplets and decreased inflammation and steatosis in hepatocytes. Compared to the high fructose group, interleukin‐1 β and tumor necrosis factor alpha were significantly decreased, serum aspartate aminotransferase concentrations were markedly reduced, and blood high‐density lipoprotein concentrations were substantially elevated after quercetin intervention (*p* < 0.05). Total bilirubin and triglyceride levels, which were significantly altered following high fructose intervention and reversed after quercetin intervention. Following the administration of 100 mg/kg quercetin, the Firmicutes/Bacteroidetes ratio was significantly reduced compared to the high fructose group. At the genus level, *Erysipelotrichaceae_uncultured*, *Faecalibaculum*, *Odoribacter*, *and Allobaculum* were significantly decreased (*p* < 0.05), *Lacnospiraceae NK4A136 group*, *Parabacteroides*, and *Alloprevotella* significantly increased (*p* < 0.05). However, the 50 mg/kg quercetin treatment only decreased the abundance of *Erysipelotrichaceae_uncultured* (*p* < 0.05). In addition, quercetin significantly enhanced the content of propionic acid and total acid (*p* < 0.05). Moreover, the intestinal flora showed a significant correlation with the hepatic health‐related phenotype in mice. Both 50 and 100 mg/kg quercetin treatments significantly mitigated liver fat deposition in mice with fructose‐induced hepatic steatosis. However, the higher dose of quercetin (100 mg/kg) demonstrated a more pronounced effect in reducing liver inflammation, likely due to its impact on gut microbiota regulation. This suggests quercetin's potential as a therapeutic agent for fructose‐related hepatic steatosis, emphasizing the importance of dose considerations.

## Graphical abstract

Quercetin can alleviate fructose‐related hepatic steatosis and inflammation in mice by regulating the composition of gut microbiota.

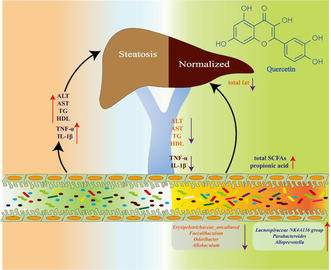

## 1 Introduction

Crystalline fructose is a hexulose isomer of glucose, the most chemically reactive six‐carbon monosaccharide sugar, and exists in a crystalline form. Due to its properties, including flavor enhancement, increased sweetness enhancement, moisture absorption, moisture retention, and a low glycemic index properties, its application in foods such as baked goods and beverages has significantly risen (Hanover and White 1993). Thus, fructose has become a crucial component of the human diet. Nevertheless, concerns about the safety of fructose have recently emerged (Zhang, Jiao, and Kong 2017). High fructose can induce non‐alcoholic fatty liver disease in mice (Chiang Morales et al. 2022). Excessive intake of fructose intake can be linked to diseases associated with liver damage‐related diseases, including elevated triglyceride (TG) levels in the blood and liver, inflammation, and oxidative stress (Kelishadi, Mansourian, and Heidari‐Beni 2014; Shi et al. 2023). Moreover, the intestinal barrier is disrupted by high fructose intake, which can lead to intestinal inflammation (Zhou et al. 2023).

Changes in the internal and surface microbial bacterial community structures have gained attention in recent years, particularly how the gut maintains normal physiological functions in the host (Zhang, Ma et al. 2022). Dietary habits greatly impact the intestinal flora composition. Quercetin is a natural bioactive flavonol polyphenol, which is one of the most bioactive polyphenols in vitro. It is found in some consumer foods including grapes, apples, onions, broccoli, and herbs (Xiao et al. 2018). Quercetin has numerous of salutary effects and biological activities, including anticancer, anti‐fatigue, antimicrobial, antioxidant, cardioprotective, anti‐inflammatory, neuroprotective, gastroprotective, and liver‐protective properties (Tsong‐Ming et al. 2015). An athletic population‐based study found that quercetin and vitamin C supplementation over 8 weeks was effective in reducing oxidative stress and inflammatory biomarkers (Askari et al. 2012). The liver–gut axis can be regulated by quercetin to improve non‐alcoholic fatty liver disease (NAFLD) (Porras et al. 2019, 2017). Because polyphenols are not easily absorbed by the digestive tract, their activity in the host is typically mediated through complex and dynamic interactions with gut microbes (Perler, Friedman, and Wu 2023). Gut microbes can convert polyphenols into their metabolites, thereby improving gut health and promoting host health (Aleksandra et al. 2015; Guilloteau et al. 2010). Increasing evidence has shown that polyphenols can be used as prebiotics to actively regulate the intestinal flora and short‐chain fatty acids (SCFAs) to promote overall host health (Zhang, Yu et al. 2022; Wang, Chen et al. 2022; Wang, Zhu et al. 2022).

Although quercetin has been reported to regulate gut microbiota in the treatment of NAFLD, there is still a lack of studies specifically examining its preventive effects against high fructose induced hepatic steatosis by quercetin (Porras et al. 2017; Yang et al. 2019). Therefore, this study primarily aimed to investigate how quercetin modulates intestinal flora to prevent high fructose‐induced hepatic steatosis.

## 2 Materials and Methods

### 2.1 Animals and Sampling

Thirty‐two 3‐week C57BL/6J male mice (18–22 g) were purchased (SLAC Laboratory Animal, Shanghai, China). This study has been approved by the Zhejiang Provincial Laboratory Animal Ethics Board (Ethical Approval: 78865576). Mice were kept in a clean environment with alternating light and dark. The indoor temperature and humidity were 22°C–24°C and 50%–80%, respectively. Mice were given unrestricted access to food and water. Crystalline fructose was purchased from Xiwang Sugar Co. Ltd. Quercetin was purchased from Ruiying Biotechnology Co. Ltd.

Grouped the mice into four groups:

1. Control group (*n* = 8): Mice received only pure water and a standard diet for 20 weeks.
2. High fructose (HFrD) group (*n* = 8): Mice were given a 30% fructose solution with their standard diet for 20 weeks.
3. HFrD +50 mg/kg BW quercetin (HFrD+LQ) group (*n* = 8): Mice were given a 30% fructose solution and 50 mg/kg BW quercetin by gavage, plus a standard diet for 20 weeks.
4. HFrD +100 mg/kg BW quercetin (HFrD+HQ) group (*n* = 8): Mice were given a 30% fructose solution and 100 mg/kg BW quercetin by gavage, plus a standard diet for 20 weeks.

The last two groups were administered quercetin for 8 weeks starting from week 12. 28 weeks later, the mice were anesthetized and dissected. The colonic contents were collected and stored at −80°C. Blood collected from mice and analyzed with an automated biochemical analyzer (Chemray240, Servicebio). Serum concentrations of alanine aminotransferase (ALT), aspartate aminotransferase (AST), low‐density lipoprotein (LDL), high‐density lipoprotein (HDL), total bilirubin (TBil), and triglyceride (TG) were identified. Tumor necrosis factor alpha (TNF‐α), interleukin‐10 (1L‐10), interleukin‐6 (1L‐6), and interleukin‐1 β (1L‐1 β) were determined with ELISA kits (Jiancheng, Nanjing, China). Body weight, liver weight, perennial fat, liver fat, and epididymal fat in mice were measured. Liver/body weight and liver fat percentage (liver fat/liver weight) were calculated.

### 2.2 Liver Histological Analysis

The Thermo Fisher NX50 freezing microtome (Thermo, USA) was used to freeze fresh liver tissues and Oil Red O was used to stain them. Using paraffin‐embedded liver tissue samples, they were sectioned and used for hematoxylin and eosin staining after being fixed in 4% paraformaldehyde. Sections were observed and photographed under an optical microscope (BX53, Olympus).

### 2.3 DNA Extraction, 16SrDNA Sequencing, and Data Analysis

Genomic DNA was extracted from the contents of the colon with the help of the QIAamp DNA Stool Mini Kit (QIAGEN) and then subjected to electrophoresis on a 1% agarose gel at 1% (Li et al. 2003). The V3‐V4 regions of the 16SrDNA gene were amplified by synthesizing specific barcoded primers based on the specified sequencing regions. The primer sequences were as follows: 515F: 5′‐GTGCCAGCMGCCGCGGTAA‐3′ and 907R: 5′‐CCGTCAATTCMTTTRAGTTT‐3′. The agarose gel was sliced to recover the amplification products, which were quantified using the QuantiFluor system and sequenced using the HiSeq 2500 PE250 platform after purification, and raw sequencing data were subjected to filtering, tag assembly, tag filtering, and removal of chimeric tags to eliminate low‐quality data and obtain effective tags for subsequent analysis. The analysis of operational classification unit (OTUs) was done on sequences that were not repeated and had a 97% similarity (Wang, Chen et al. 2022; Wang, Zhu et al. 2022). Species matching was performed for all representative sequences of the OTUs using the RPD databases (https://rdp.cme.msu.edu/).

### 2.4 SCFAs Analysis

The concentration of SCFAs in the sample was detected by gas chromatography. The concentrations of acetic acid, propionic acid, butyric acid, and total acid in the samples were detected by gas chromatography (GC‐2010 Plus; Shimadzu Corporation, Kyoto, Japan), and the column was DB‐FFAP type (Agilent Technologies Inc., Santa Clara, CA, USA). with hydrogen flame ionization detector and crotonic acid as internal standard. As shown in Figure S2, we constructed standard curves for acetic acid, propionic acid, and butyric acid. The corresponding equations and correlation coefficients (*R* ^2^) are provided in the figure. These standard curves can be used to calculate the concentration of SCFAs in the samples based on their peak areas.

### 2.5 Statistical

SPSS 26.0 software was utilized to analyze and and graphs were plotted using GraphPad Prism 8 software. The mean and standard error of the mean were used to compare differences using analysis of variance. The data for 16SrDNA were mapped using the R language. Bacterial taxa are distinguished by the size of the effect of linear discriminant analysis (LDA) using a linear discriminant score threshold. Principal component analysis (PCA) and Principal Coordinates Analysis (PCoA) was used to analyze α and β diversity among the samples. One‐way ANOVA was used for microbiota compositions and the content of SCFAs. Pearson linear correlation analysis and redundancy analysis (RDA) were employed to examine the connection between intestinal microbiota and disease phenotypes. The statistical significance of a *p*‐values < 0.05 was considered.

## 3 Results

### 3.1 Quercetin Administration Prevents Hepatic Steatosis Induced by High‐Fructose Diet

**FIGURE 1:**
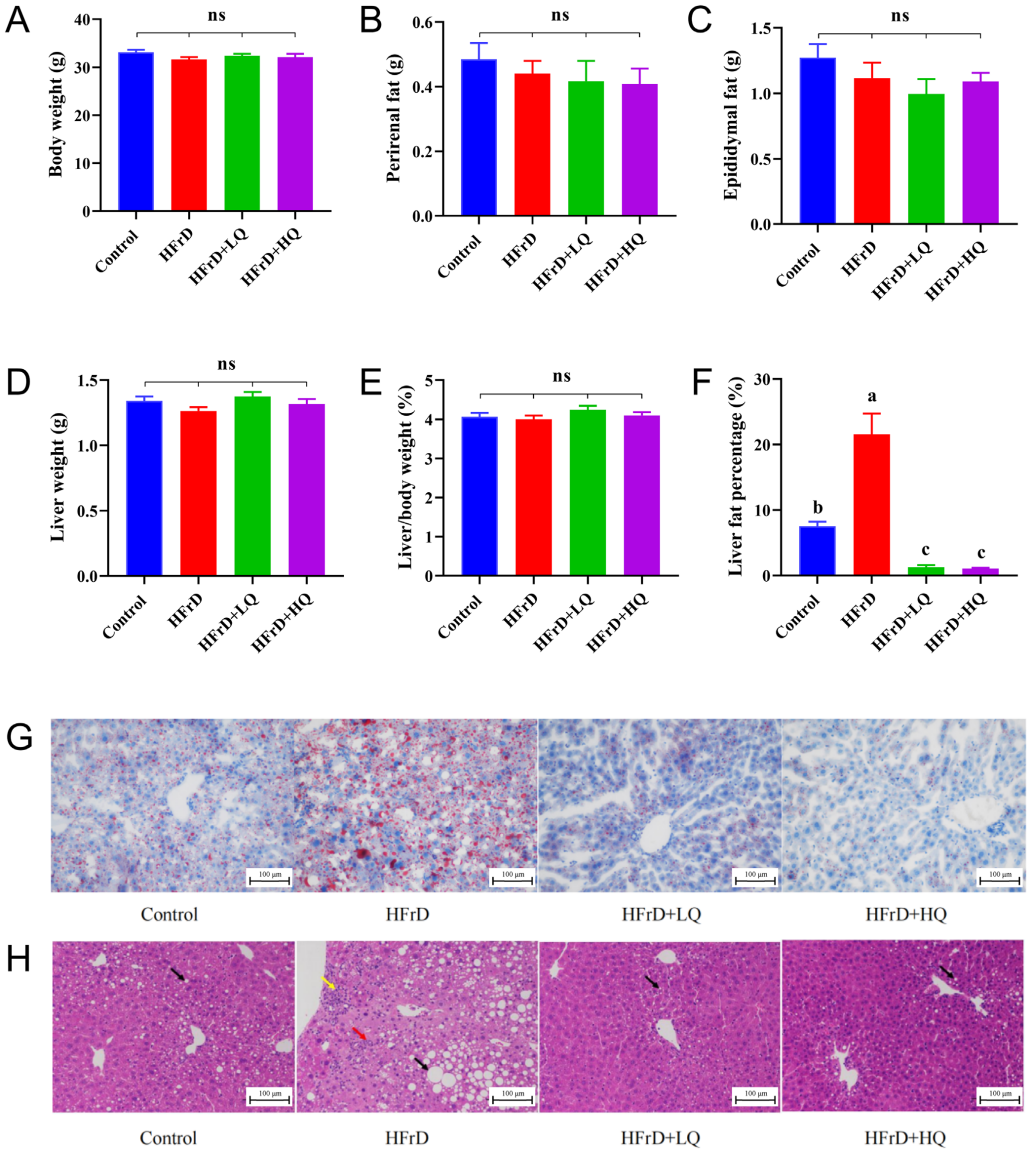
Phenotypic data characterization (*n* = 8). (A–F) Body weight, perirenal fat, epididymal fat, liver weight, liver body weight, and liver fat percentage, respectively. Different lowercase letters denote a significant difference (*p* < 0.05), with the same letter indicating no significant difference (*p* > 0.05). Histological analysis of tissues dissected from the four groups of mice (*n* = 3). (G) Frozen liver sections stained with Oil Red O after dissection from the four groups. (H) Liver tissue sections stained with hematoxylin and eosin. The bar on each micrograph is 100 μm. Black arrows show rounded vacuoles of hepatocyte steatosis, yellow arrows show spotty infiltration of lymphocytes, and red arrows show bile duct hyperplasia in the portal area. “ns” indicates no significant difference in the indicator across all groups.

As shown in Figure 1A–E, there were no significant differences in the body weight, perirenal fat weight, epididymal fat weight, liver weight, and liver/body weight ratio among the control, HFrD, HFrD+LQ, and HFrD+HQ groups. The liver fat percentage of the HFrD group was significantly higher than that of the control group (*p* < 0.05). After treatment with quercetin at doses of 50 and 100 mg/kg BW, the liver fat percentage in the mice was significantly lower than that in the HFrD group (*p* < 0.05) and even lower than that in the control group (*p* < 0.05) (Figure 1F).

As shown in Figure 1G, Oil red O staining of the liver sections indicated that the lipid droplets in the HFrD group was higher than that in the control group. The content of lipid droplet content in the HFrD group was significantly reduced following intervention with 50 and 100 mg/kg BW of quercetin intervention, as shown in Figure 1H. Hematoxylin and eosin staining revealed an obvious circular cavity of hepatic steatosis (black arrow), infiltration of spotted lymphocytes (yellow arrow), and bile duct hyperplasia in the portal area (red arrow) in the livers of HFrD group mice treated with 50 and 100 mg/kg BW of quercetin. The hepatopathy was significantly reduced, with only a small amount of liver fat was observed as deformed round vacuoles (black arrow).

### 3.2 High‐Dose Quercetin Treatment Protects Mice From Liver injury Induced by Crystalline Fructose

**FIGURE 2:**
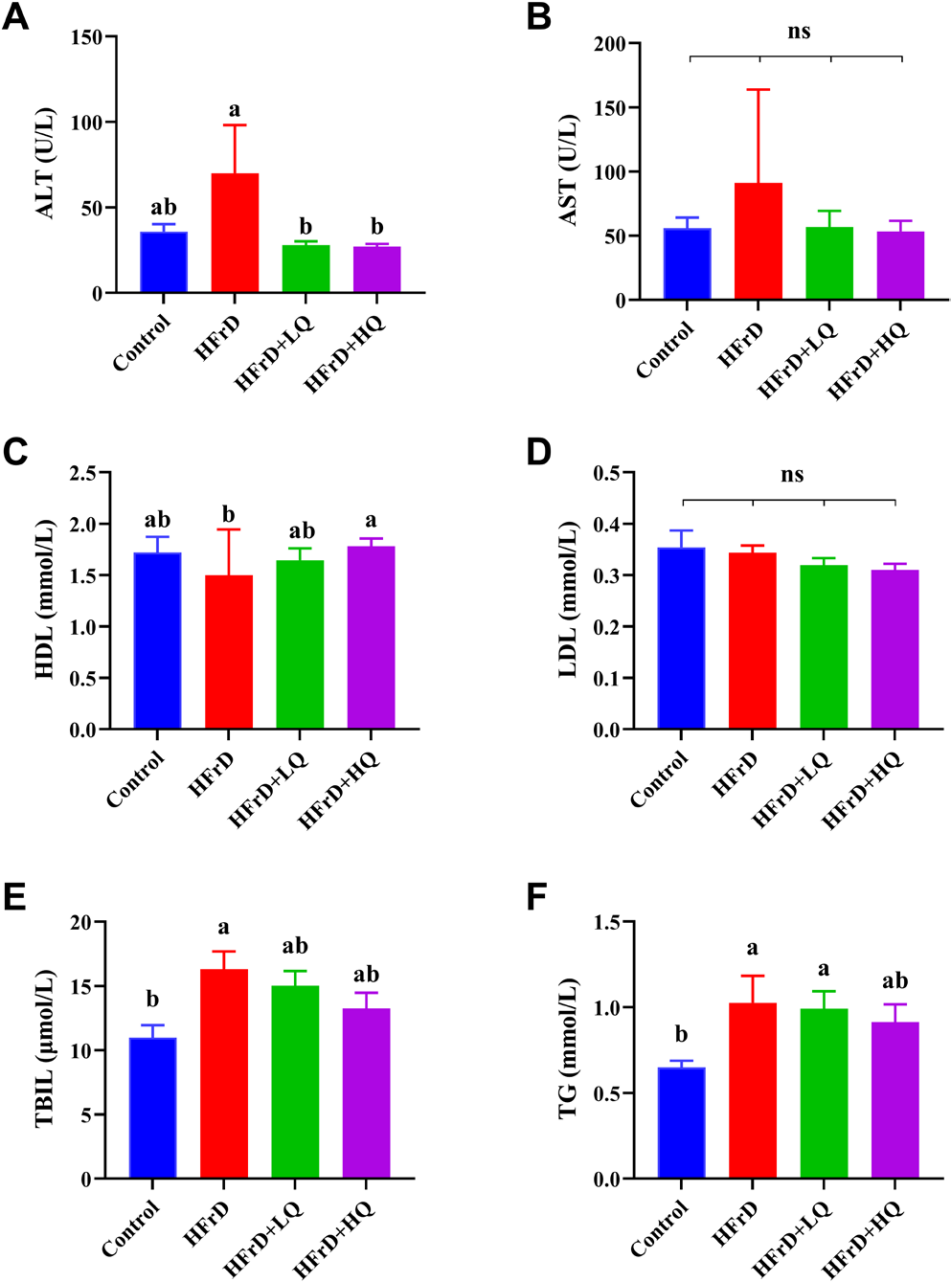
Liver Function‐Related Indicators (*n* = 8). (A–F) ALT, AST, HDL, LDL, TBIL, and TG, respectively *n* = 8. Different lowercase letters indicate a significant difference (*p* < 0.05), while the same letter indicates no significant difference (*p* > 0.05). “ns” indicates no significant difference in the indicator across all groups.

As shown in Figure 2A, quercetin intervention significantly reversed the increase in ALT levels induced by crystalline fructose. (*p* < 0.05). The trend in the changes of serum AST levels corresponded with the trend observed in ALT levels, although no significant differences were detected between them (*p* > 0.05) (Figure 2B). There was no significant difference in HDL levels among the control group, HFrD, and the HFrD+LQ groups; however, in the HFrD+HQ group, HDL levels were significantly higher than those in the other three groups (*p* < 0.05). As shown in Figure 2D, the LDL content did not significantly differ among the control, HFrD, HFrD+LQ, and HFrD+HQ groups. The TBIL (total bilirubin) content in the HFrD group was significantly higher than that in the control group (*p* < 0.05). Additionally, the TBIL contents in the HFrD+LQ and HFrD+HQ groups tended to decrease compared to those in the HFrD and control groups, but the differences were not statistically significant (Figure 2E). As shown in Figure 2F, the TG content in the HFrD group was significantly greater than that in the control group. Although there were no significant differences between the quercetin treatment groups and the HFrD group, the high dose of quercetin reduced TG levels to a level similar to the control group (*p* > 0.05).

### 3.3 High‐Dose Quercetin Treatment Protects Mice From Inflammation Induced by Crystalline Fructose

**FIGURE 3:**
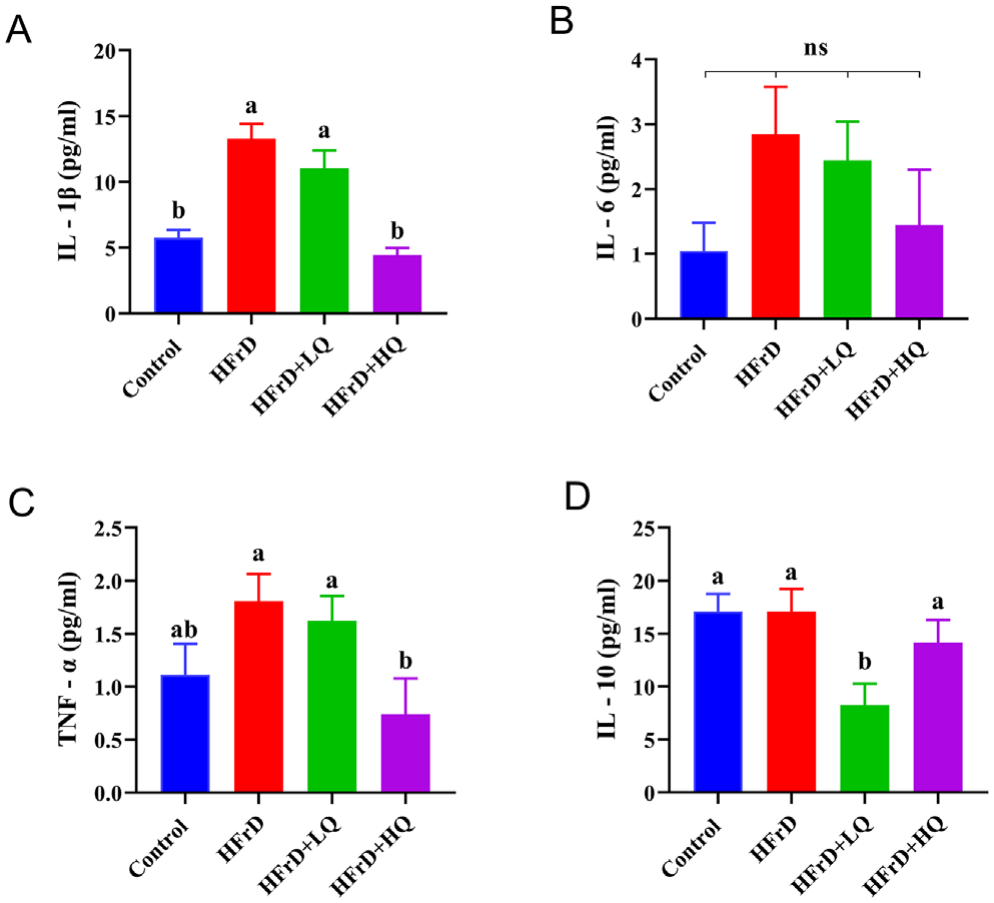
Effects on Blood Inflammatory Indices in Mice (*n* = 8). (A–D) Levels of inflammatory cytokines IL‐1β, IL‐6, TNF‐α, and IL‐10 in the serum, respectively. Different lowercase letters indicate significant differences (*p* < 0.05), while the same letter indicates no significant difference (*p* > 0.05). “ns” indicates no significant difference in the indicator across all groups.

As shown in Figure 3A–D, after 28 weeks of high fructose solution feeding, the expression of the inflammatory cytokines IL‐1β, IL‐6, and TNF‐α in the HFrD group was higher than that in the control group. Following the quercetin intervention trial, the expression of IL‐1β, IL‐6, TNF‐α, and IL‐10 was decreased compared to that in the HFrD group. Notably, IL‐1β and TNF‐α levels in the HFrD+HQ group and IL‐10 levels in the HFrD+LQ group were significantly lower than those in the HFrD group (*p* < 0.05).

### 3.4 Effects of High Fructose Solution and Quercetin Administration of Gut Microbiota Composition

#### 3.4.1 Differences in Microbiota Compositions in Mice

The distribution of the four groups of intestinal microflora can be seen in Figure S1. In this pie chart, the Firmicutes/Bacteroidetes ratio was reduced in the high fructose solution group compared with the control. Combined with Figures S1 and 4, at the phylum level, the dominant bacterial communities were Bacteroidetes, Firmicutes, Proteobacteria, Tenericutes, Deferribacteres, and Cyanobacteria, accounting for more than 99.46% of the colonic microflora in mice. Compared with the control group, the relative abundance of Bacteroidetes decreased significantly and that of Firmicutes increased significantly in the HFrD group (*p* < 0.05 for both). In contrast, in the HFrD+HQ group, the abundance of Bacteroidetes increased significantly compared to the crystal fructose group (*p* < 0.05), while the abundance of Firmicutes decreased significantly (*p* < 0.05). Combined with Figures S1 and 5, we further analyzed changes in the colonic microbiota composition at the genus level. The top 10 genera in relative abundance of intestinal microbiota in all groups were *Bacteroidales S24‐7 group_norank*, *Allobaculum*, *Erysipelotrichaceae_uncultured*, *Alistipes*, *Faecalibaculum*, *Bacteroides*, *Lachnospiraceae NK4A136 group*, *Parabacteroides*, *Odoribacter*, and *Rikenellaceae RC9* gut groups. Among these, compared with the HFrD group, after quercetin intervention, *Erysipelotrichaceae_uncultured*, *Faecalibaculum*, and *Odoribacter* were significantly decreased (*p* < 0.05), while *Allobaculum*, *Lachnospiraceae NK4A136* group, and Parabacteroides were significantly increased (*p* < 0.05).

**FIGURE 4:**
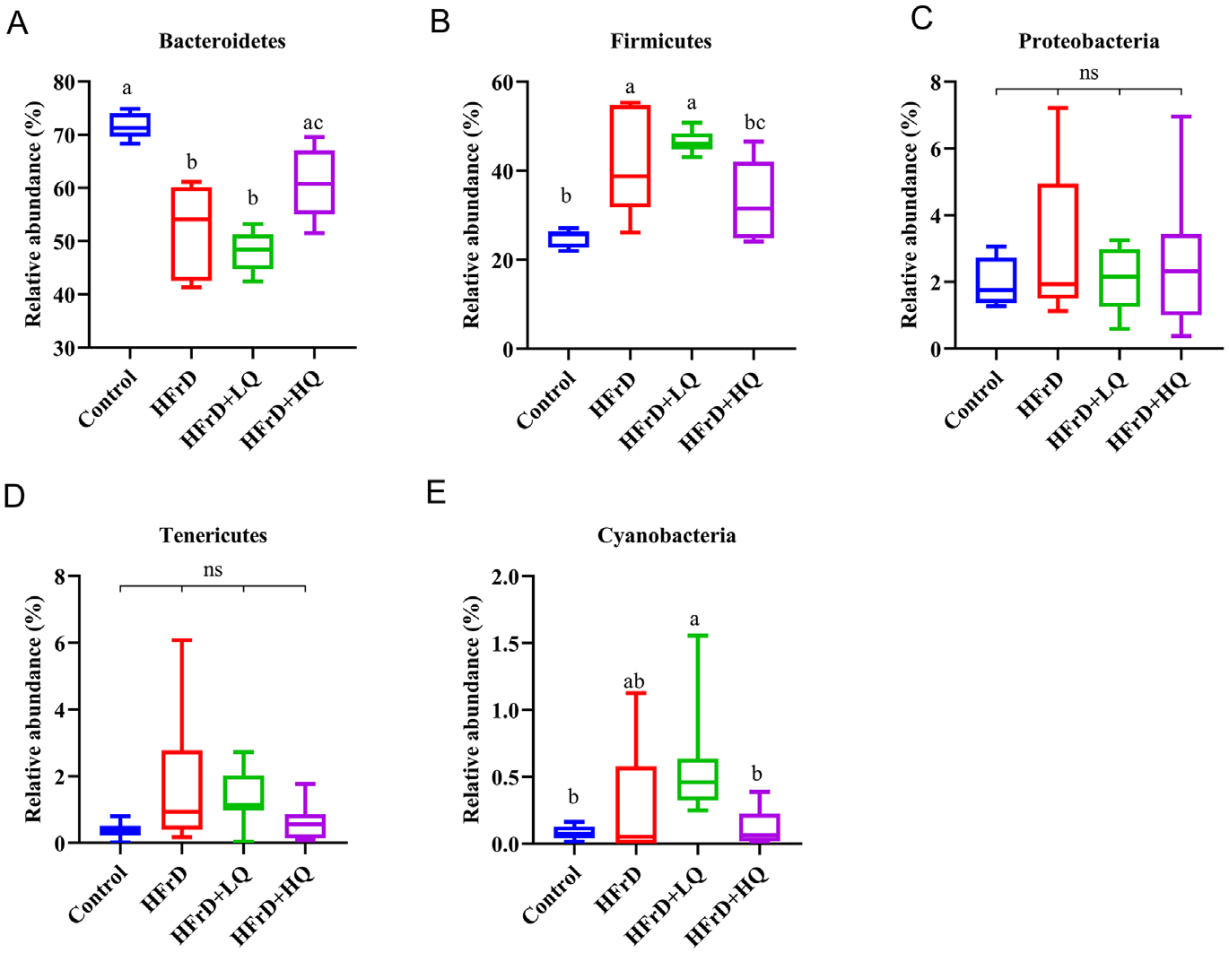
Analysis of Differences at the Phylum Level (A–E) (*n* = 8). Different lowercase letters indicate significant differences (*p* < 0.05), while the same letter indicates no significant difference (*p* > 0.05). “ns” indicates no significant difference in the indicator across all groups.

**FIGURE 5:**
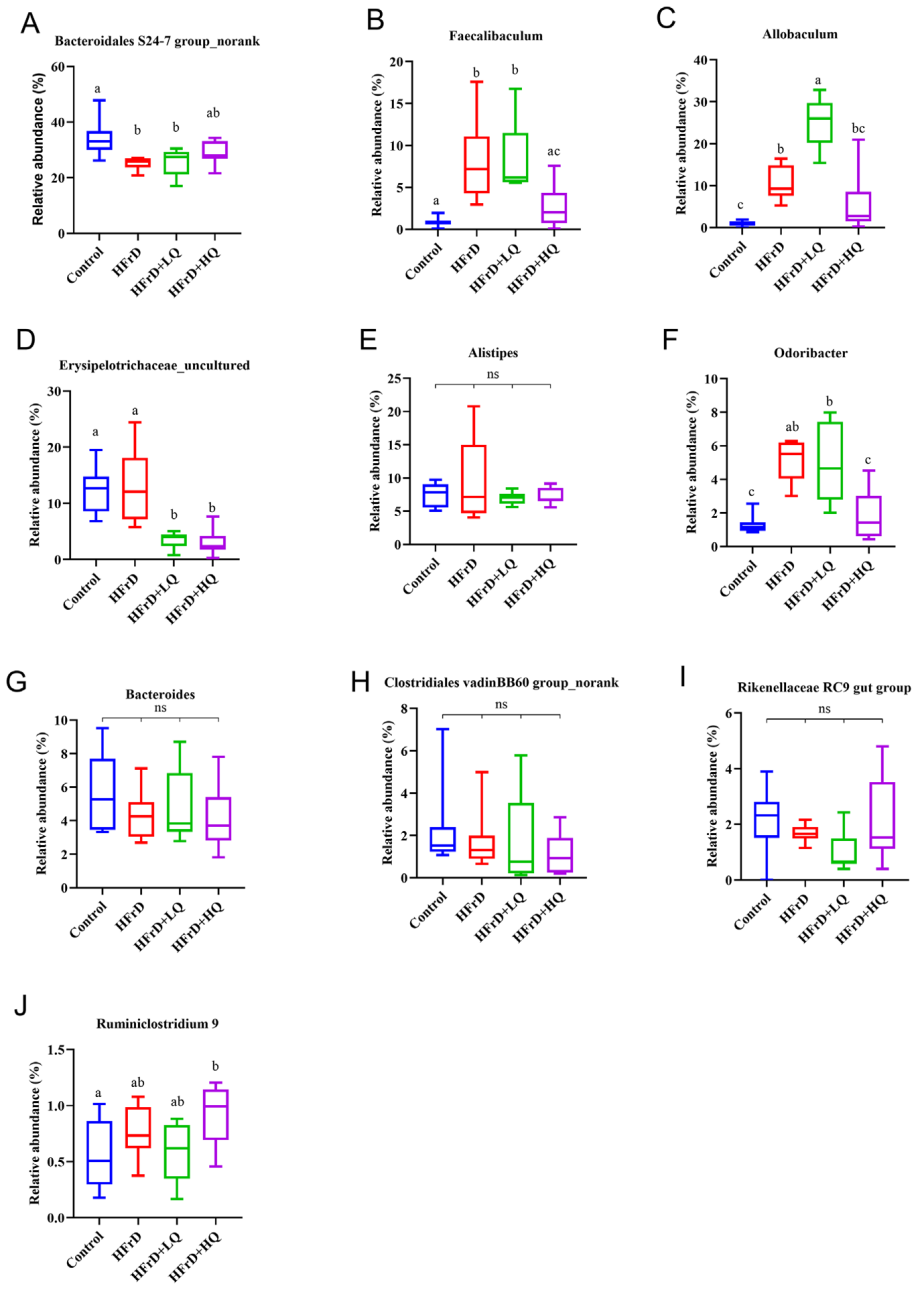
Analysis of differences at the genus level (A–J) (*n* = 8). Different lowercase letters indicate significant differences (*p* < 0.05), with the same letter indicating no significant difference (*p* > 0.05). “ns” indicates no significant difference in the indicator across all groups.

**FIGURE 6:**
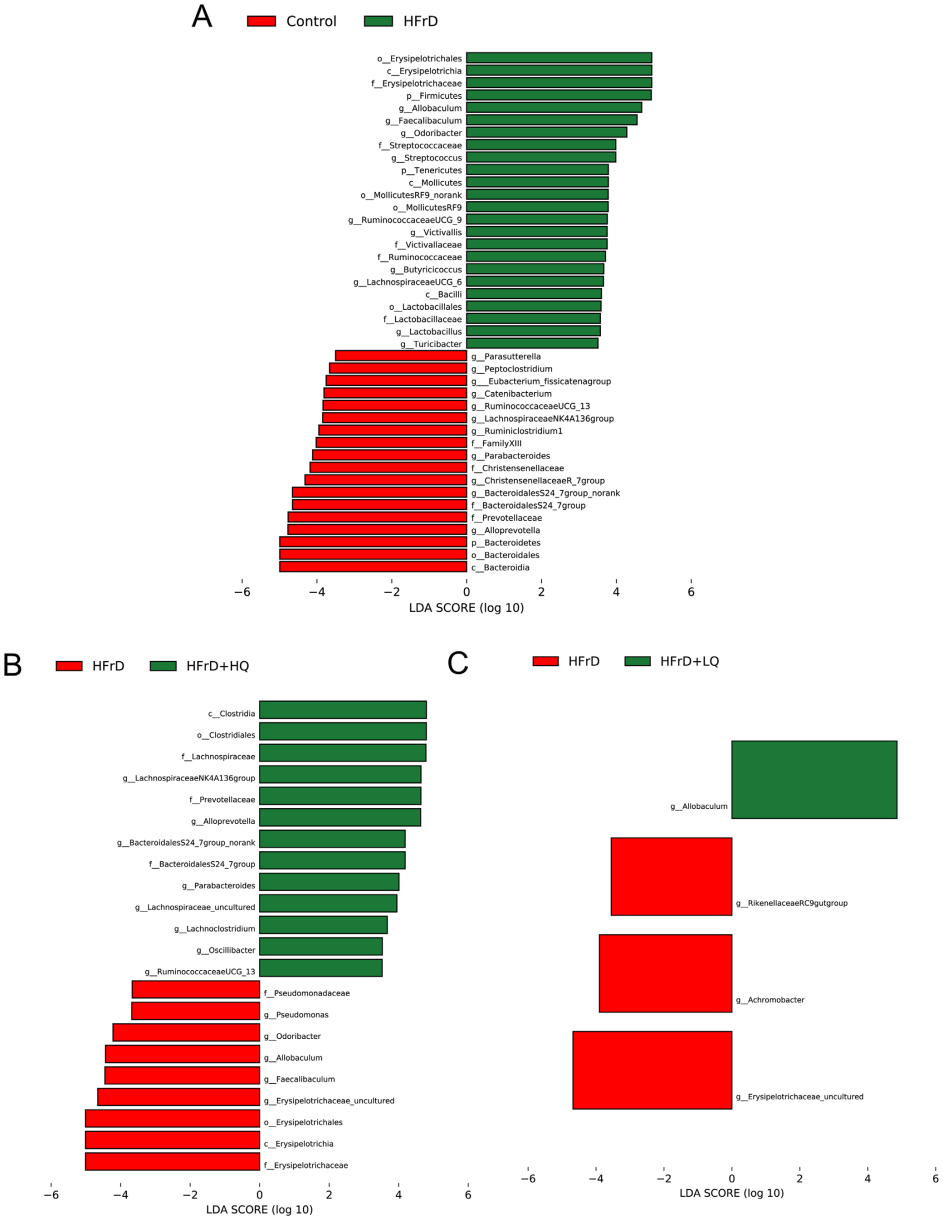
Bacterial taxa differentially represented in the colon of mice in the control, HFrD, HFrD + LQ, and HFrD + HQ groups were identified using linear discriminant analysis effect size with a linear discriminant analysis (LDA) score threshold of > 2.5. *n* = 8.

To detect enrichment of specific bacterial taxa among the four groups, we performed linear discriminant analysis (LDA) effects size (LDA score threshold of > 3.5, *p* < 0.05), which emphasizes statistical significance and biological consistency. As shown in Figure 6, in contrast to the control group, the HFrD group experienced a decline in the abundance of several bacterial taxa, including *Alloprevotella*, *Bacteroidales S24_7 group*, *Parabacteroides*, *Lachnospiraceae NK4A136*, and *Ruminococcaceae UCG_13*. Concurrently, there was an increase in the abundance of *Odoribacter* and *Allobaculum* within this group. Intriguingly, in the HFrD+HQ group, the abundance patterns of these genera were reversed, indicating a restorative effect. In contrast, the low‐dose BW quercetin treatment did not significantly alter the abundance of these microbial taxa.

#### 3.4.2 Alpha and Beta Diversity of Colon Flora in Mice

**FIGURE 7:**
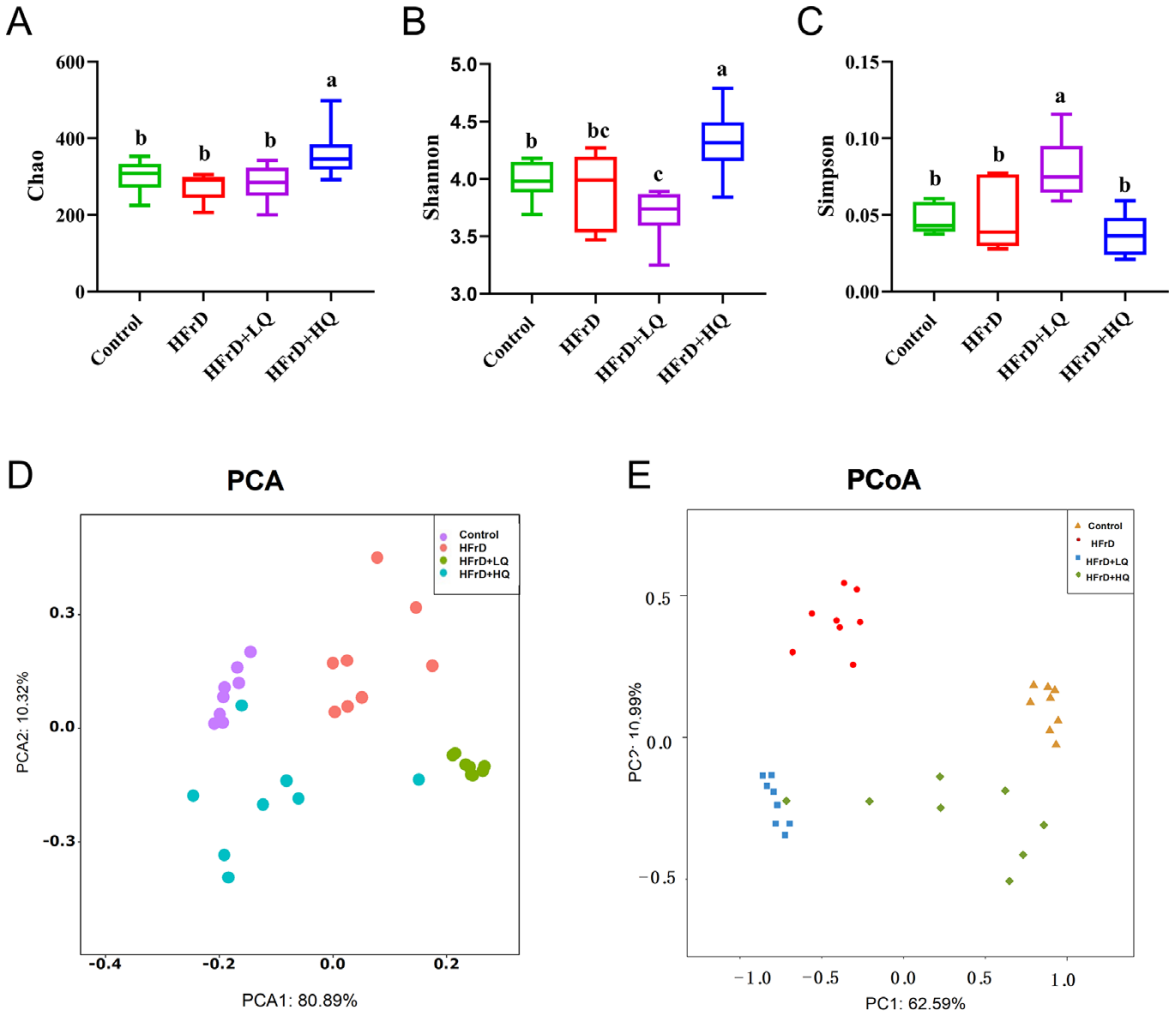
Alpha diversity. (A–C) Chao, Shannon, and Simpson indices, respectively. Beta diversity. (D, E), Principal Coordinate Analysis (PCA) and Principal Component Analysis (PCoA) of the bacterial structure of the mouse cecum. *n* = 8. Different lowercase letters indicate a significant difference (*p* < 0.05), with the same letter indicating no significant difference (*p* > 0.05).

The Chao, Shannon, and Simpson indices of the HFrD group showed no significant difference compared to the control group, as shown in Figure 7A–C. After treatment with quercetin, the Chao index of the HFrD+HQ group was significantly higher than that of the HFrD group (*p* < 0.05), and the Shannon and Simpson indices in the HFrD+HQ group were significantly higher (*p* < 0.05) and lower (*p* < 0.05), respectively, than those in the HFrD group. These results indicate that the diversity and abundance of colonic microflora were improved in HFrD+HQ group mice.

As shown in Figure 7D,E, Principal Component Analysis (PCA) and Principal Coordinates Analysis (PCoA) revealed the presence of significantly different clusters among the control, HFrD, HFrD+LQ, and HFrD+HQ groups, suggesting that high fructose solution altered the intestinal flora structure in mice, which was also altered after treatment with high and low doses of quercetin.

### 3.5 Differences in Short‐Chain Fatty Acids (SCFAs) Among the Four Groups

**FIGURE 8:**
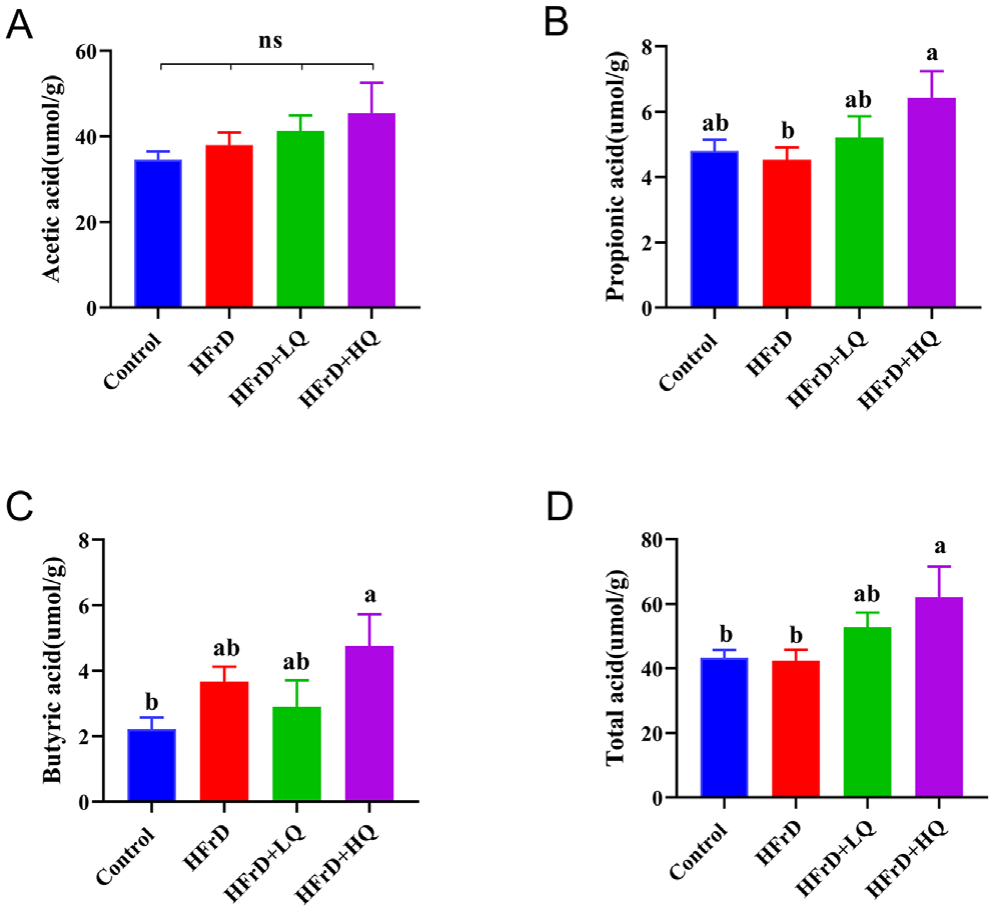
Differences in levels of short‐chain fatty acids among the four groups (*n* = 8). (A–D) Levels of total, acetic, propionic, and butyric acids, respectively. Different lowercase letters indicate a significant difference (*p* < 0.05), while the same letter indicating no significant difference (*p* > 0.05). “ns” indicates no significant difference in the indicator across all groups.

As shown in Figure 8, using gas chromatography to detect short‐chain fatty acids (total acid, acetic acid, propionic acid, and butyric acid), we found no significant difference in the content of acetic acid among the four groups. Compared with the control group, HFrD group had no significant difference in total acid, propionic acid, and butyric acid. Total acid and propionic acid contents in HFrD+HQ group were significantly higher than those in HFrD group (*p* < 0.05), butyric acid content was also higher than that in HFrD group, but the difference was not significant (*p* = 0.67). In conclusion, the most significant increase in propionic acid and total acid content was observed after high‐dose quercetin intervention.

### 3.6 The Relationship Between Bacterial Community and Inflammation and SCFAs

**FIGURE 9:**
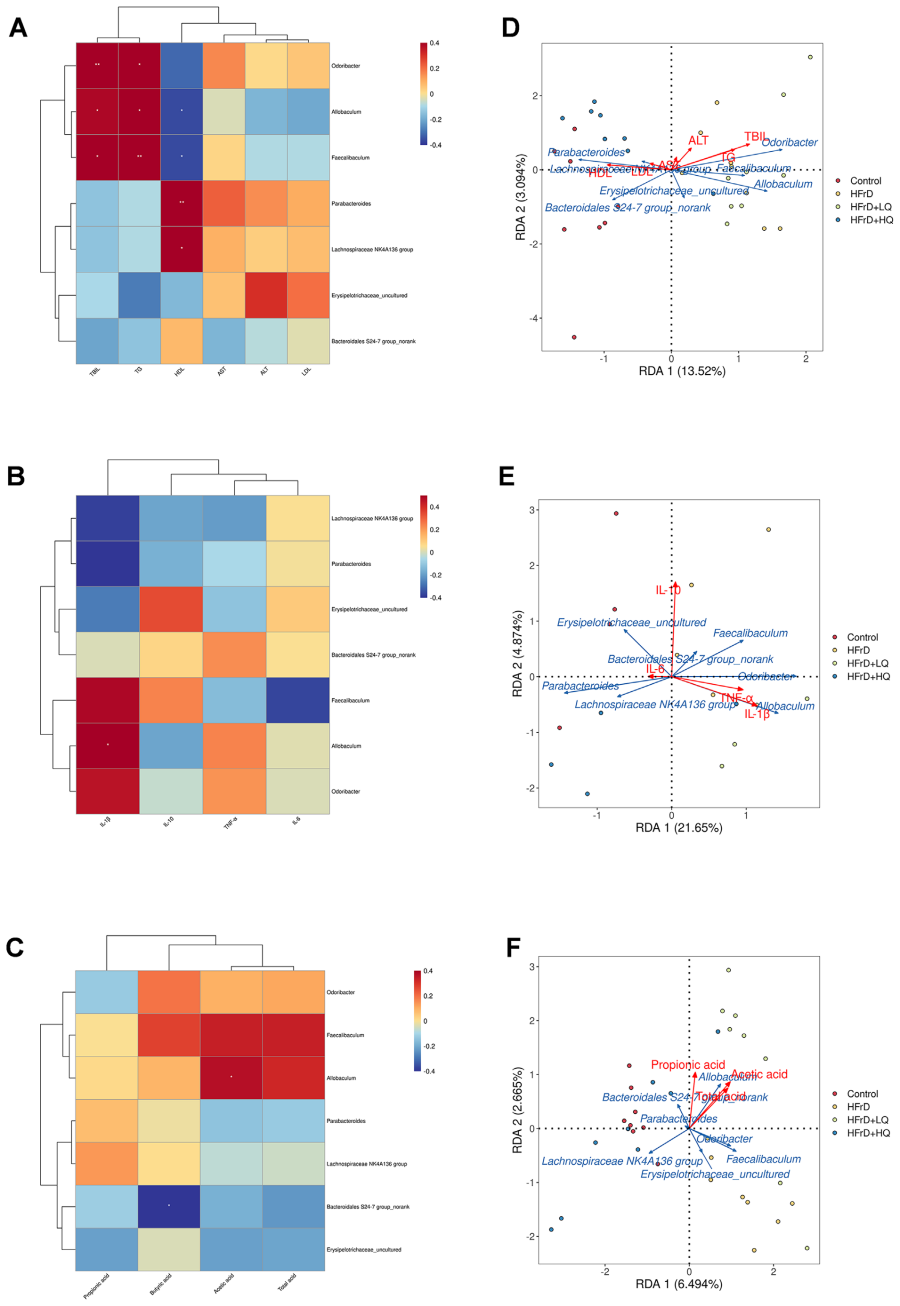
Correlation analysis and Redundancy Analysis (RDA). Correlation analysis between liver function indexes and dominant flora (A). Correlation analysis of short‐chain fatty acids with dominant flora (B). Correlation analysis between inflammatory factors and dominant flora (C). (D–F) shows the RDA analysis microbiota and liver indicators, microbiota and inflammatory factors, and between microbiota and SCFAs, respectively. In this figure, blue arrows indicate the microbiota and red arrows indicate the other detection indicators. The longer the arrow, the greater the correlation; the shorter the arrow, the weaker the correlation. The Angle between the arrow line and the sorting axis and the Angle between the arrow line represents the correlation. The acute Angle represents the positive correlation, and the obtuse Angle represents the inverse correlation. The smaller the Angle, the higher the correlation. *n* = 8. *p < 0.05; **p < 0.05.

As shown in Figure 9A–C, the results from the hepatic index correlation analysis revealed a significant positive correlation between TBIL and TG and *Odoribacter*, *Allobaculum*, and *Faecalibaculum*. HDL was negatively correlated with *Allobaculum* and *Faecalibaculum* (*p* < 0.05), and positively correlated with *Parabacteroides* and *Lacnospiraceae NK4A136* group (*p* < 0.05). The correlation analysis for inflammatory indices revealed a significant positive correlation between Il‐1β and *Allobaculum* (*p* < 0.05). Correlation analysis of short‐chain fatty acids showed that butyric acid was significantly negatively correlated with *Bacteroidales S24‐7_group no rank* and acetic acid was significantly positively correlated with *Allobaculum* (*p* < 0.05).

Moreover, we conducted an RDA Analysis on each index and dominant flora (Figure 9D–F). As shown in Figure 9D, TBIL, TG, and HDL had the longest line with the origin, and the RDA index was close to 1, indicating that these three indices had the highest correlation with flora. The longest lines were *Odoribacter*, *Allobaculum*, and *Parabacteroides*. Among them, TBIL and TG were positively correlated with *Odoribacter* and *Allobaculum*, but negatively correlated with *Parabacteroides*. HDL was negatively correlated with *Odoribacter* and *Allobaculum* and positively correlated with *Parabacteroides*. As shown in Figure 9E, 1L‐10 and 1L‐1β had the longest line with respect to the origin, and the RDA index was close to 1, indicating that these two indices had the greatest correlation with the flora. *Allobaculum*, *Odoribacter*, and *Parabacteroides* were the most significant flora. 1L‐1β was positively correlated with *Allobaculum* and *Odoribacter* and adversely correlated with *Parabacteroides*. A negative correlation was detected between 1L‐10 and *Allobaculum* and Parabacteroides, and a positive correlation was detected between 1L‐10 and *Odoribacter*. As shown in Figure 9F, the line between propionic acid and the origin is near 1, and the correlation with bacteria is the greatest. The most different flora were Erysipelotrichaceae_uncultured, the *Lacnospiraceae NK4A136 group*, and *Allobaculum*. *Allobaculum* was positively correlated with propionic acid, *Erysipelotrichaceae_uncultured*, and the *Lacnospiraceae NK4A136 group* was negatively correlated with propionic acid.

In conclusion, reduced levels of *Odoribacter*, *Allobaculum*, and *Faecalibaculum* were associated with lower TBIL and TG levels, while increased *Parabacteroides* and *Lacnospiraceae NK4A136* populations corresponded to higher HDL content. A decrease in *Allobaculum* and *Faecalibaculum* increased the HDL content. A decrease in *Allobaculum* can significantly increase the content of acetic acid and decrease the content of 1 L‐1β. The addition of the *nonranked Bacteroidales S24‐7_group* increased the propionic acid content.

## 4 Discussion

As one of the main flavonol polyphenols. Polyphenols also regulate the composition of the intestinal flora. Quercetin was found to decrease the ratio of Firmicutes/Bacteroidetes and the relative abundances of *Firmicutes*, *Erysipelotrichia*, and *Bacillus* in both animals and humans. *Bacillus*, *Eubacterium*, *Cylindroides*, and quercetin‐liposomes have been shown to improve liver damage caused by amoxicillin/clavulanate by targeting SIRT1/Nrf2/NF‐κB and modifying the microbiota, as demonstrated in the present study. *Ruminococcus1* and *Streptococcus* were significantly more abundant in the HFrD+LQ group than that in the control and HFrD groups. The proportion of Firmicutes/Bacteroidetes in the HFrD+HQ group was significantly lower than that in the HFrD group. The relative abundance of *Erysipelotrichaceae* in the HFrD group was high but was significantly decreased after quercetin intervention, which is in line with the results of previous studies. In a fecal microbiota (Cui et al. 2022), a clinical trial demonstrated that reduction of *Erysipelotrichaceae* could alleviate liver degeneration in fatty liver (Jinato et al. 2022). Reversing the increased proportion of *Erysilotrichaceae* can improve glucose and lipid metabolism disorders caused by liver injury in mice. *Faecalibaculum* is a flora that is abundant in the human gut and decreases in different disease states (Leylabadlo et al. 2020). The decrease of *Odoribacter* can maintain intestinal balance and reduce the occurrence of constipation (Du et al. 2022). Thus, at the genus level, the ability of high‐fructose solution to reverse dysbacteriosis caused by high fructose solution may involve reducing the abundance of *Allobaculum*, *Erysipelotricha‐ceae_uncultured*, *Faecalibaculum*, and *Odoribacter* and increasing the abundance of the *Lacnospiraceae NK4A136 group*, *Parabacteroides*. A high‐sugar diet has been reported to promote obesity in many studies (Tappy and Le 2010; Turnbaugh et al. 2009), and the increase in *Firmicutes* to *Bacteroidetes* abundance is thought to be related to body fat deposition. Fructose has a significant effect on the microbiota of rodents, and mice‐fed fructose had a significant decrease in the abundance of Bacteroidetes, whereas the abundance of Firmicutes was slightly increased 44 and have a significant decrease in the abundance of Bacteroidetes, whereas the abundance of Firmicutes slightly increases. Quercetin has strong pharmacological activity in the treatment of liver diseases, including hepatic steatosis, steatohepatitis, hepatic fibrosis, and liver cancer. It can significantly affect the development of liver disease through multiple targets and pathways involved in anti‐fat accumulation, anti‐inflammatory and antioxidant activities, and inhibition of apoptosis and proliferation (Zhao et al. 2021). Animal studies have shown that quercetin protects mice and rats from weight gain and adipose tissue accumulation induced by a high‐fat diet (Zhao et al. 2017). Evidence from clinical studies revealed that quercetin consumption had a significant effect on total fat content and body mass index in overweight and obese individuals (Luo et al. 2023). Quercetin also reduced the waist fat accumulation around the waist and TG concentrations in overweight individuals and individuals with various apolipoprotein E genotypes (Zhao et al. 2017). Quercetin reversed the fatty liver induced by high‐fructose solution in mice, as we previously reported. Both the 50 and 100 mg/kg BW doses of quercetin reduced the liver fat percentage and improved the characteristics of liver cells in the mice fed with the high‐fructose diet, with the best effects observed in the HFrD+HQ group. HFrD+HQ reduced both the IL‐1β and TNF‐α levels in the livers of the mice, whereas HFrD reduced the IL‐10 levels.

In addition to dietary fiber, plant‐derived polyphenols, which are catabolic in the intestinal microbial ecosystem, are important substrates of the intestinal microbiota and may affect the intestinal microecosystem (Zhang et al. 2023). An animal experiment has shown that most quercetin is metabolized in the intestine, especially in the colon, and that only a small amount is metabolized in the liver (Xiao et al. 2005). The intestinal flora enhances the bioactivity of polyphenols by converting them into active metabolites; *Bifidobacterium*, *Lactobacillus*, *Escherichia coli*, *Bacteroides*, *Eubacterium*, *Enterococcus*, *Bifidobacterium*, *Ruminococcus*, *Streptococcus S‐2*, and other bacteria can catalytically metabolize phenols via catabolism (Lan et al. 2023). The role of the intestinal microbiota in nonalcoholic fatty liver disease (NAFLD) has been confirmed in a wide range of studies (Kolodziejczyk et al. 2018; Leung et al. 2016). Quercetin can be used to treat NAFLD because of its ability to regulate intestinal microbiota disorders and the related enterohepatic axis. Quercetin can regulate the intestinal flora, exert anti‐inflammatory and antioxidant effects, reduce the liver fat content, and maintain the body's steady state in patients with NAFLD (Petrov et al. 2019). Quercetin liposomes have been shown to improve liver damage caused by amoxicillin/clavulanate by targeting SIRT1/Nrf2/NF‐κB and modifying the microbiota, as demonstrated in studies (Abd El‐Emam et al. 2023). Among them, the key role of the intestinal flora could involve lowering the ratio of Firmicutes/Bacteroidetes and increasing the abundance of Proteobacteria (Cheng et al. 2022). Supplementation with quercetin significantly increased the abundance of *Sutterella*, *Allobaculum*, and *Flavobacterium* and reduced the relative abundance of *Helicobacter* and *Desulfovibrio*. The increase in *Firmicutes* to *Bacteroidetes* abundance is thought to be related to body fat deposition (Tappy and Le 2010; Turnbaugh et al. 2009). Fructose has a significant effect on the microbiota of rodents, and mice‐fed fructose presented a significant decrease in the abundance of Bacteroidetes, whereas the abundance of Firmicutes slightly increased (Beisner et al. 2020). As shown in our study, after treatment with 100 mg/kg BW quercetin, but not 50 mg/kg BW quercetin, the Firmicutes/Bacteroidetes ratio in the intestines of HFrd mice was significantly restored.

The intestinal microbiota is an important factor affecting body fat deposition (Woods, Nehra, and Vandana 2016; Zamparelli et al. 2016). Quercetin was also found to decrease the relative abundance of *Erysipelotrichaceae* in both animals and humans. *Erysipelotrichaceae* were previously shown to reduce inflammation, insulin resistance, and adipogenesis caused by high‐fat and high‐sugar diets (Etxeberria et al. 2015; Shi et al. 2020). A clinical trial demonstrated that a reduction in *Erysipelotrichaceae* could alleviate liver degeneration in fatty liver (Jinato et al. 2022). Reversing the increased proportion of *Erysilotrichaceae* can improve glucose and lipid metabolism disorders caused by liver injury in mice. In our study, the relative abundance of *Erysipelotrichaceae* in the HFrD group was high and significantly decreased after quercetin intervention, which is in line with the results of previous studies. *The fecal microbiome* is a flora that is abundant in the human gut and decreases in different disease states (Leylabadlo et al. 2020). A decrease in *Odoribacter* can maintain the intestinal balance and reduce the occurrence of constipation (Du et al. 2022). ANGPTL4 is a key regulator of lipid metabolism and a circulating medium for the gut microbiota and fat deposition, and the *Allobaculum* was shown to be positively correlated with ANGPTL4 expression in high‐fat diet‐fed mice (Zheng et al. 2021). In addition, the abundance of Allobaculum was increased in NAFLD model mice (Ni et al. 2023) and *Allobaculum* was also enriched in nonalcoholic steatohepatitis model rats. In this study, *Erysipelotrichaceae_uncultured*, *Faecalibaculum*, *Allobaculum*, and *Odoribacter* were significantly enriched in the HFrD group. Both the low‐dose and high‐dose quercetin treatments decreased the abundance of *Erysipelotrichaceae_uncultured*, but the abundances of *Faecalibaculum*, *Allobaculum*, and *Odoribacter* were decreased only in the HFrD+HQ group. In a fecal microbiota transplantation experiment, the addition of *the Lachnospiraceae NK4A136 group* was proven to treat fatty liver disease (Pande et al. 2023). There is a strong association between high‐fat and high‐sugar diets and *Parabacteroides* (de Wouters d'Oplinter et al. 2021). *Parabacteroides* has a potential therapeutic role in maintaining host‐gut homeostasis (Cui et al. 2022) and has been confirmed to play a positive role in alleviating NAFLD and obesity (Li et al. 2023). Previous studies have shown that increased *Alloprevotella* can help alleviate liver damage in both alcoholic liver disease (ALD) (Guo et al. 2022) and NAFLD mice (Mao et al. 2024). We found that the *Lacnospiraceae NK4A136 group*, *Parabacteroides*, and *Alloprevotella* were elevated in the HFrD+HQ group; however, low‐dose quercetin treatment did not affect the abundance of these bacteria. Therefore, we hypothesized that, compared with those in the HFrD+LQ group, the lower levels of inflammation exhibited in the HFrD+HQ group may be related to its regulatory effect on the gut microbiota.

Additionally, following quercetin intervention, the HFrD+LQ and HFrD+HQ groups presented significant increases in some bacteria that produce anti‐inflammatory and short‐chain fatty acids, including *Allobaculum*, *Faecalibaculum*, *Lactobacillus*, *Coprococcus*, *Butyricicoccus*, and *Blautia*. Short‐chain fatty acids inhibit inflammation and obesity (Guilloteau et al. 2010). Acetic acid, propionic acid, and butyric acid are produced by the intestinal flora, which metabolize carbohydrates (Cummings 1984). Stewart et al. (2008) reported that quercetin decreased the expression of inflammatory markers in mice. The propionic acid content in the HFrD‐HQ group was significantly greater than that in the HFrD group in this study. The butyric acid content was also higher than that in the HFrD group, but not significantly. Low‐ and high‐dose quercetin significantly reduced the IL‐10 and IL‐1β levels, respectively. These results demonstrated that quercetin may increase the anti‐inflammatory effect by increasing the content of short‐chain fatty acids.

In summary, long‐term consumption of a high‐fructose solution caused no significant changes in the final body weights of the mice. However, the consumption of this water resulted in fatty deposition in the livers of the mice; changes in the intestinal flora, including increases in the genera *Erysipelotrichaceae* and *Clostridium*; and increases in the ratio of *Firmicutes* to *Bacteroidetes*. The relative abundance of some beneficial bacteria with anti‐inflammatory and short‐chain fatty acid production effects, such as *Allobaculum*, *Faecalibaculum*, *Lactobacillus*, *Coprococcus*, *Butyricicoccus*, and *Blautia*, was increased significantly. Quercetin can significantly reverse the damage caused by high‐fructose solutions to the liver and regulate the structure of the intestinal flora in mice.

## 5 Conclusion and Limitations

We systematically explored the effects of quercetin on high‐fructose‐induced hepatic steatosis by combining biochemical, pathological, immune, and metabolic data. The composition of the gut microbiota and liver fat deposition can be altered by long‐term fructose intake, as we discovered. By regulating the abnormal gut microbiota caused by fructose intake, 100 mg/kg quercetin can promote host health. Among these, the gut microbiota decreases the IL‐1β and TNF‐α levels effect and favors the production of propionic acid and total acid, thus playing a protective role in the liver. Unfortunately, to date, this study has focused only on mice. If quercetin is to better fulfills its pharmaceutical role, not only further exploration of its safe dose but also additional clinical trials are necessary.

## Author Contributions

**Jinjun Li:** conceptualization (equal), data curation (equal), formal analysis (equal), funding acquisition (equal), methodology (equal), project administration (equal), writing – original draft (equal), writing – review and editing (equal). **Zhiqi Zhao:** investigation (equal), writing – review and editing (equal). **Yixuan Deng:** investigation (equal), writing – review and editing (equal). **Xinxin Li:** investigation (equal), writing – review and editing (equal). **Liying Zhu:** conceptualization (equal), methodology (equal). **Xin Wang:** conceptualization (equal), methodology (equal). **Li Li:** conceptualization (equal), investigation (equal), project administration (equal). **Xiaoqiong Li:** conceptualization (equal), data curation (equal), formal analysis (equal), funding acquisition (equal), methodology (equal), project administration (equal), writing – original draft (equal).

## Ethics Statement

This study was approved by the Zhejiang Provincial Ethics Committee for Laboratory Animals (Ethical Approval No. 78865576).

## Conflicts of Interest

The authors declare no conflicts of interest.

## Supporting information


**Figure S1.** Pie charts of the distribution of colonic microbial communities in the four groups of the phylum and genus levels.
**Figure S2**. The standard curves for each single acid of SCFAs.

## Data Availability

The authors declare that the data supporting the findings of this study are presented within the manuscript. The sequences obtained in the study were deposited in the NCBI Sequence Read Archive under accession number PRJNA970969. Additional data sources are also available from the corresponding author upon reasonable request.
