# Supplementary material for: Regulatory Roles of Quercetin in Alleviating Fructose‐Induced Hepatic Steatosis: Targeting Gut Microbiota and Inflammatory Metabolites
Source: Food Sci Nutr. 2024 Dec 20;13(1):e4612. doi: 10.1002/fsn3.4612 (PMC11717000; doi:10.1002/fsn3.4612)
Supplement: Supplementary file 1 — Figure S1. Pie charts of the distribution of colonic microbial communities in the four groups of the phylum and genus levels. Figure S2. The standard curves for each single acid of SCFAs. [file FSN3-13-e4612-s001.docx]

**Supplementary_information**


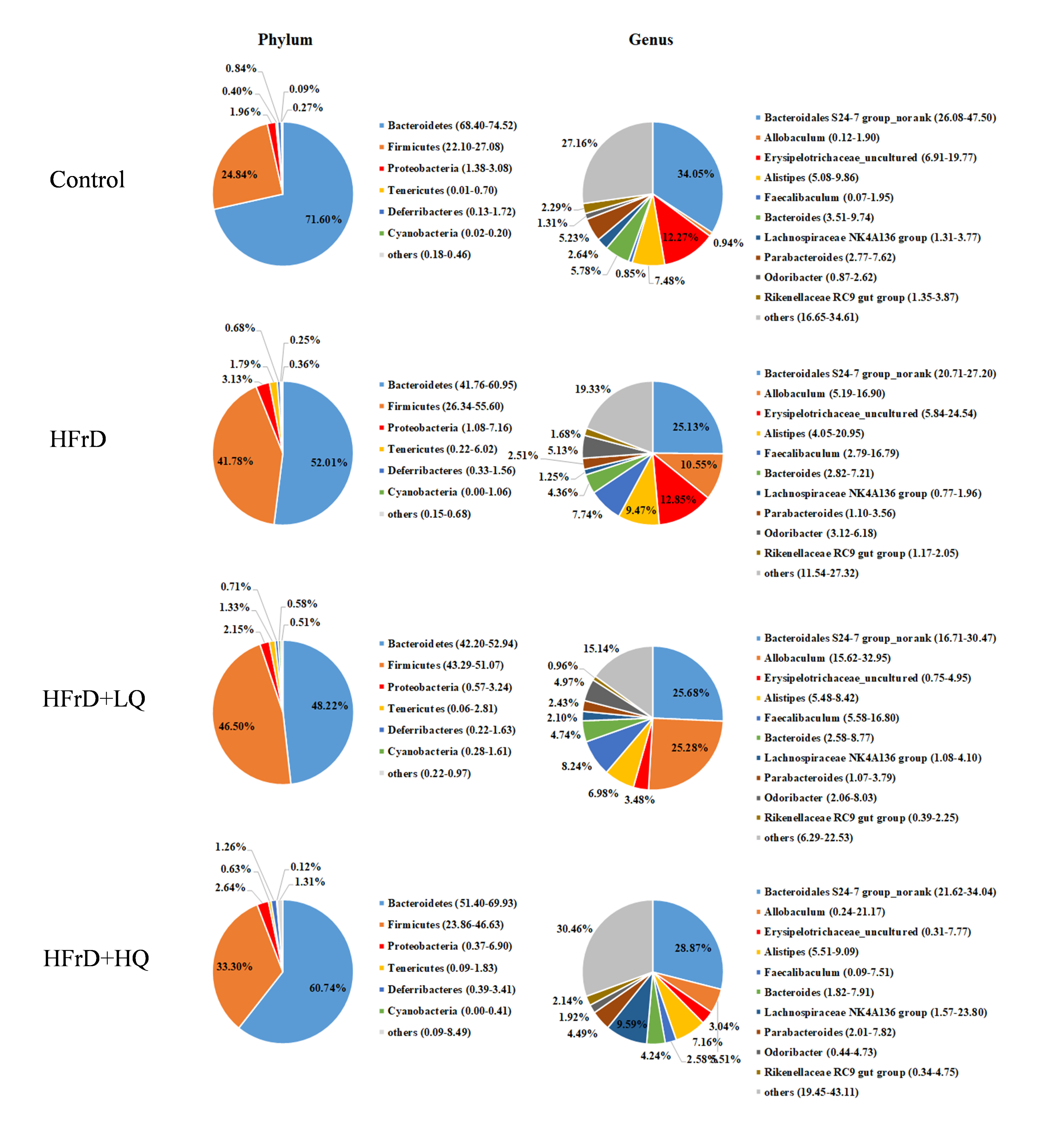


**Figure S1.** Pie charts of the distribution of colonic microbial communities in the four groups of the phylum and genus levels. Only the top six phyla and top 10 genera with the highest relative abundances are shown.


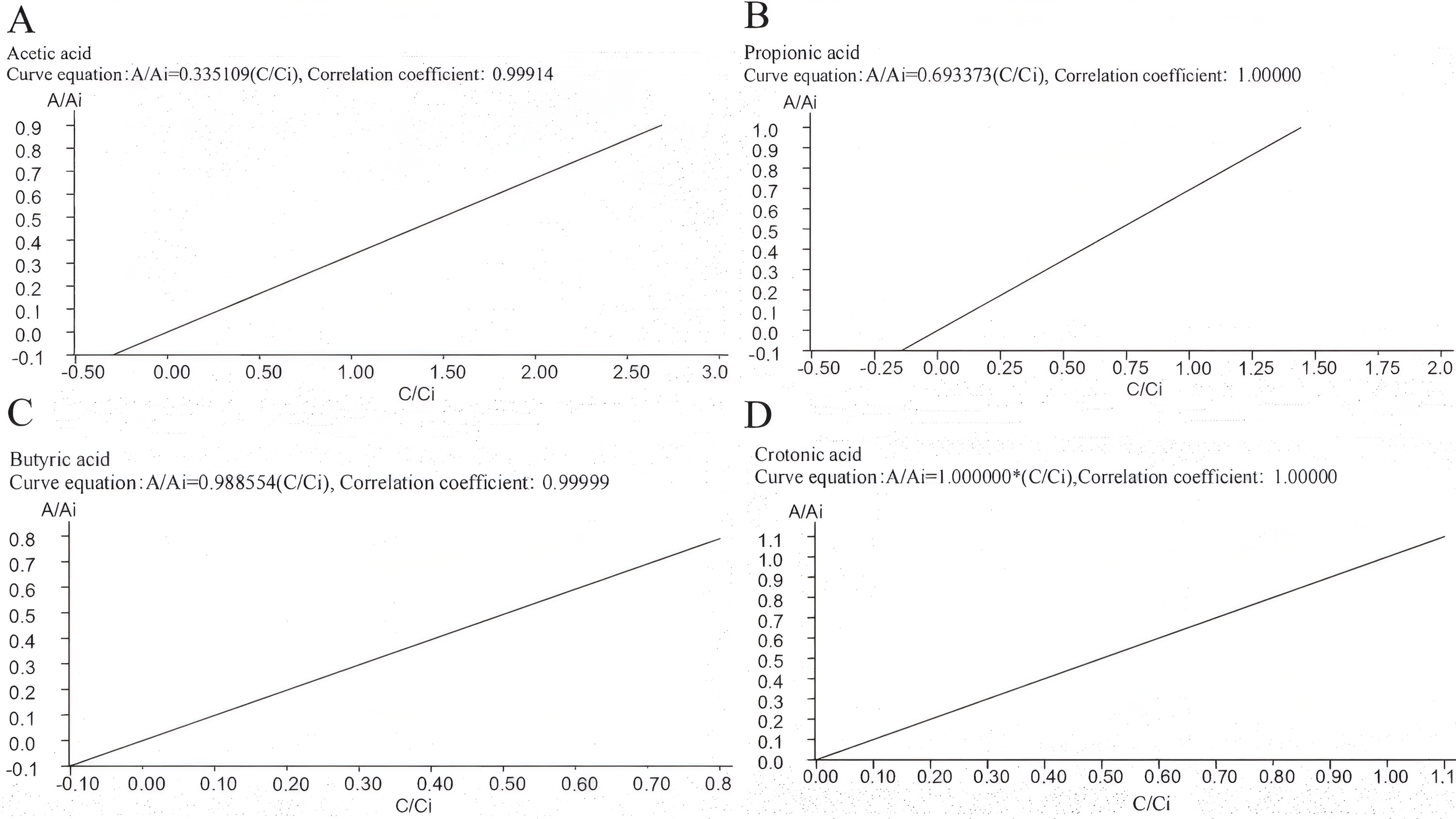


**Figure S2.** The standard curves for each single acid of SCFAs. Acetic acid (A), Propionic acid (B), Butyric acid (C), Crotonic acid (D).
